# Applying a random encounter model to estimate lion density from camera traps in Serengeti National Park, Tanzania

**DOI:** 10.1002/jwmg.902

**Published:** 2015-05-28

**Authors:** Jeremy J Cusack, Alexandra Swanson, Tim Coulson, Craig Packer, Chris Carbone, Amy J Dickman, Margaret Kosmala, Chris Lintott, J Marcus Rowcliffe

**Affiliations:** 1 Department of Zoology, University of Oxford, The Tinbergen BuildingSouth Parks Road, Oxford OX1 3PS, United Kingdom, and Institute of Zoology, Zoological Society of London, London NW1 4RY, United Kingdom; 2 Department of Ecology, Evolution and Behavior University of MinnesotaMN, 55108, USA; 3 Department of Zoology, University of OxfordOxford OX1 3PS, United Kingdom; 4 Department of Ecology, Evolution and Behavior, University of MinnesotaMN, 55108, USA; 5 Institute of Zoology, Zoological Society of LondonLondon NW1 4RY, United Kingdom; 6 Department of Zoology, University of OxfordOxford OX1 3PS, United Kingdom; 7 Department of Ecology, Evolution and Behavior University of MinnesotaMN, 55108, USA; 8 Department of Physics, University of OxfordOxford OX1 3RH, United Kingdom; 9 Institute of Zoology, Zoological Society of LondonLondon NW1 4RY, United Kingdom

**Keywords:** camera traps, density estimation, habitat, lion, *Panthera leo*, random encounter model, REM, Serengeti

## Abstract

The random encounter model (REM) is a novel method for estimating animal density from camera trap data without the need for individual recognition. It has never been used to estimate the density of large carnivore species, despite these being the focus of most camera trap studies worldwide. In this context, we applied the REM to estimate the density of female lions (*Panthera leo*) from camera traps implemented in Serengeti National Park, Tanzania, comparing estimates to reference values derived from pride census data. More specifically, we attempted to account for bias resulting from non-random camera placement at lion resting sites under isolated trees by comparing estimates derived from night versus day photographs, between dry and wet seasons, and between habitats that differ in their amount of tree cover. Overall, we recorded 169 and 163 independent photographic events of female lions from 7,608 and 12,137 camera trap days carried out in the dry season of 2010 and the wet season of 2011, respectively. Although all REM models considered over-estimated female lion density, models that considered only night-time events resulted in estimates that were much less biased relative to those based on all photographic events. We conclude that restricting REM estimation to periods and habitats in which animal movement is more likely to be random with respect to cameras can help reduce bias in estimates of density for female Serengeti lions. We highlight that accurate REM estimates will nonetheless be dependent on reliable measures of average speed of animal movement and camera detection zone dimensions. © 2015 The Authors. *Journal of Wildlife Management* published by Wiley Periodicals, Inc. on behalf of The Wildlife Society.

Camera traps are used worldwide to answer a range of questions relevant to ecology and conservation (O’Connell et al. [Bibr b24], Meek et al. 2014). A common aim of many camera trap surveys is estimating the density of a target species within an area of interest. To this end, recent spatially explicit capture-recapture (SECR) methods, which combine both the spatial and temporal information contained in photographs of recognizable individuals, have provided unbiased estimates of density for marked species, i.e., those for which animals are individually recognizable (Borchers and Efford 2008, Royle et al. 2009, Gardner et al. 2010, Gopalaswamy et al. 2012). Spatially explicit capture-recapture analysis is now supported by a substantial body of literature mainly focusing on spotted and striped felids (Sollmann et al. 2011, Gray and Prum 2012, Athreya et al. 2013). In contrast, no well-established methods exist for estimating the density of unmarked species using camera traps (Carbone et al. 2001, Jennelle et al. 2002, Chandler and Royle 2013), despite these representing the majority of species likely to be photographed (Tobler et al. 2008).

Rowcliffe et al. (2008) proposed a random encounter model (REM) that describes the rate of contact between moving animals and static camera traps to estimate species density. The REM requires a species encounter rate (sensu Carbone et al. 2001) to be estimated, along with a camera detection zone specified by its radius and angle, and an estimated average speed of movement for the target species. A key assumption of the model is that cameras are placed randomly with respect to animal movement (Rowcliffe et al. 2013), meaning that they should not be targeted so as to inflate, or deflate, encounter rates. Studies using the REM have been implemented by deploying cameras in systematic or fully randomized arrays (Rovero and Marshall 2009, Manzo et al. 2012), with camera placement determined a priori by precise geographical coordinates rather than influenced by the presence of features that may increase capture probability (Rowcliffe et al. 2008, Rovero et al. 2013).

To date, however, the REM has never been used to estimate the density of a large carnivore species (Foster and Harmsen 2012). We applied the model to estimate the density of female lions (*Panthera leo*) from an extensive camera trap survey implemented in Serengeti National Park, Tanzania, comparing estimates to reference values derived from pride census data (Schaller 1972, Bygott et al. 1979, Packer et al. 2005). As a threatened and unmarked large carnivore for which reliable estimates of abundance or density are difficult to obtain (Ogutu et al. 2006, Funston et al. 2010, Durant et al. 2011, Groom et al. 2014), the lion represents a suitable candidate species on which to test the REM. Indeed, given the decline of the species across Africa over the past decades (Riggio et al. 2013), it has become important to test the reliability of potential density estimation methods.

Importantly, the Serengeti camera trap survey was not designed with the REM in mind. Despite the use of a gridded design, the low tree density encountered in grassland habitat resulted in cameras often being placed on isolated trees whose shade attracted lions during the day, thus representing a potential violation of the random placement assumption. We assessed the effect of this known source of bias by comparing the accuracy of REM estimates derived from night-only versus all photographs, between wet and dry seasons, and across habitat types that differed in their amount of tree cover. During the night, during the wet season, and in more densely wooded habitat, lions are less likely to seek shade under trees, and we expected camera placements on trees to be closer to random with respect to lion movement. We anticipated that REM estimates derived from data filtered by these factors would show improved accuracy when compared to reference densities.

## STUDY AREA

**Figure 1:**
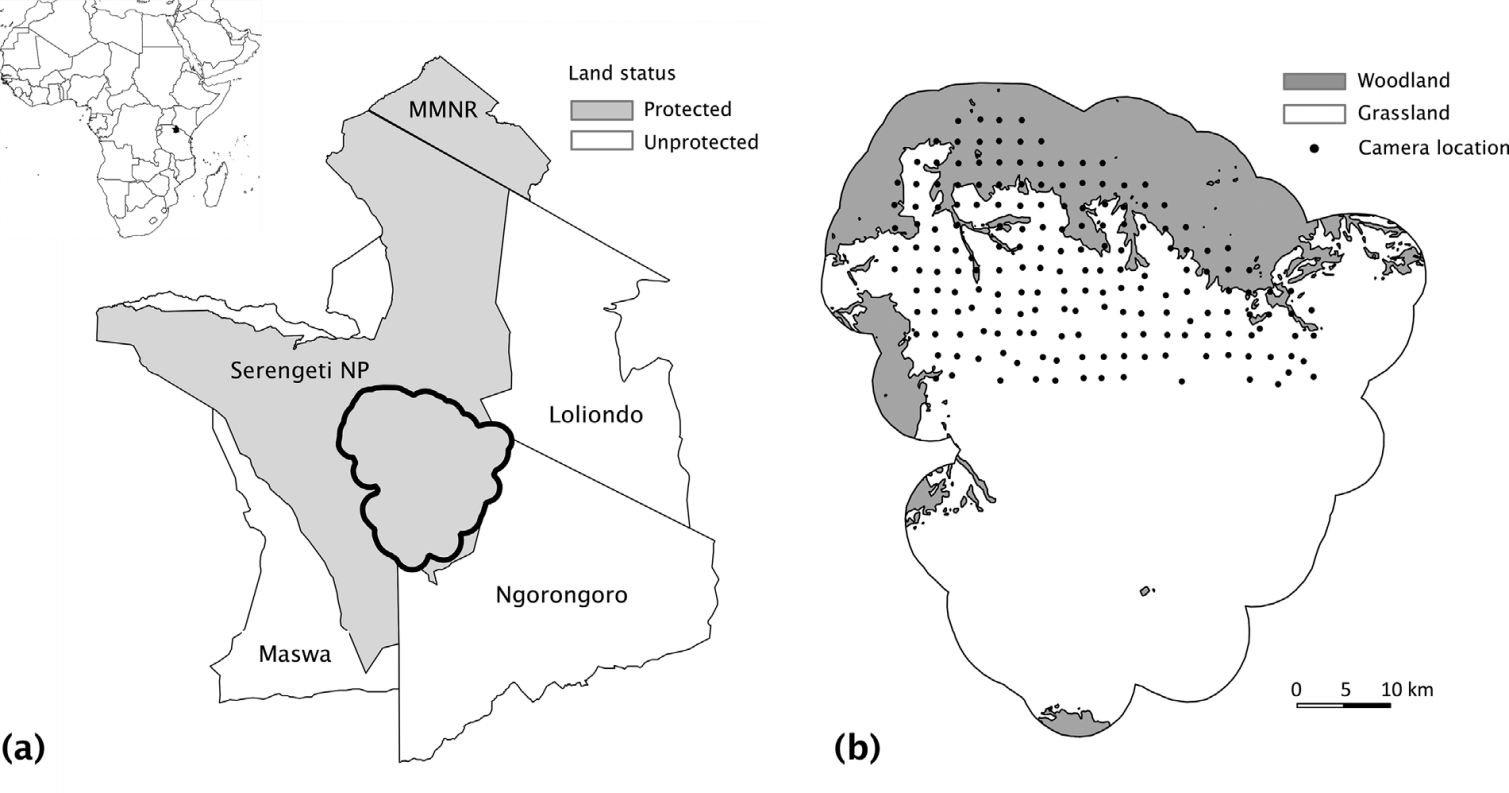
(a) Location of the Serengeti Lion Project study area (black outline) in northern Tanzania and (b) layout of the camera trap grid across woodland and grassland habitats. The Serengeti–Mara ecosystem encompasses key protected lion populations in Serengeti National Park (NP) and the Masai Mara National Reserve (MMNR) in Kenya.

The study area encompasses 1,125 km^2^ of the Serengeti National Park, Tanzania, and was located within the 25,000-km^2^ Serengeti–Mara ecosystem (Fig. 1). It is marked by a southeast-to-northwest gradient of rainfall and soil type (Norton-Griffiths 1975), which creates a transition from short-grass plains in the southeast (hereafter, grassland) to woodlands in the north (Packer et al. 2005). Lion density is largely limited by food availability in the dry season when prey biomass is at an annual low (Schaller 1972, Bertram 1975, Packer et al. 2005).

Lions live in gregarious groups known as prides, which are composed of related females, their dependent offspring, and 1 or more males (Scheel and Packer 1991, Packer et al. 2005). The latter form coalitions that can reside in, and distribute their time across, more than 1 pride (Schaller 1972, Bygott et al. 1979). In contrast, nomads do not maintain a territory and move great distances through the ecosystem (Schaller 1972). The population has been monitored continuously since 1974 (Bygott et al. 1979, Packer et al. 2001), and several prides have been monitored since 1966 (Schaller 1972). Since 1984, 1 female member of each study pride was radio-collared, with all subsequent monitoring relying on a combination of radio telemetry and opportunistic sightings (Mosser et al. 2009).

## METHODS

### Camera Trap Survey

The data used in this study form part of an on-going camera trap survey implemented by the Serengeti Lion Project (SLP; Swanson et al. 2015). We consider 2 3-month blocks, 1 in the dry season of 2010 (Aug–Oct) and 1 in the wet season of 2011 (Mar–May), during which 168 and 167 camera locations were defined as active, respectively (Fig. 1). We deployed camera traps (ScoutGuard SG595, Boly Media Communications, Santa Clara, CA) at the center of 5-km^2^ grid cells, resulting in an average spacing of 2.3 km. This cell size aimed to ensure a minimum number of 5 traps per pride home range. In the case of the REM, spatial autocorrelation between neighboring cameras is not considered a problem because the approach focuses on contact rates between cameras placed randomly with reference to animal movement. We used a handheld global positioning system (GPS) device to locate cell centroids and placed each camera trap on the closest tree within a 1-km radius of the corresponding point. If no trees were located within that distance, we attached cameras to man-made poles (8.7% of camera placements).

Camera settings were chosen as part of a large-scale, multi-species survey and were not specific to lions (Swanson et al. 2015). During the dry season, camera traps were programmed to take a sequence of 3 pictures per trigger during the day and at night. During the wet season, cameras took only 1 picture per trigger at night. We stress that this difference is unlikely to have resulted in bias because female lions were observed in the first of 3 photographs in 98.6% of events taken during the day. As a result, the number of pictures per trigger is unlikely to influence lion detection probability. In both seasons, there was an arbitrary 1-minute delay between consecutive triggers. The date and time of capture were automatically stamped onto each image. Although the camera model used an incandescent flash for night-time pictures, it is unlikely to have modified the behavior of lions, which in the Serengeti are habituated to humans and research equipment.

We initially processed camera trap images to extract and quality control date and time metadata. We then imported the images into the Snapshot Serengeti (see www.snapshotserengeti.org) citizen science website for content classification. The latter combined multiple classifications of each image to yield high accuracy determinations of species (see Swanson et al. 2015 for more details).

### Reference Densities

We calculated reference densities for female lions in grassland and woodland habitats for both the dry season of 2010 and the wet season of 2011. Habitat delineation was based on a classification of Landsat images (LPDAAC, USGS/EROS, Sioux Falls, SD) into 24 vegetation assemblages by Reed et al. (2009) using the method put forward by Grunblatt et al. (1989). The Serengeti GIS and Data Centre later grouped these assemblages into 4 broad vegetation types based on percentage tree canopy cover (51–100% = dense woodland/forest; 21–50% = open woodland; 2–20% = savannah; less than 2% = grassland; available at www.serengetidata.org). In this study, we combined the dense woodland/forest and open woodland categories to define woodland polygons, and used the categories grassland and savannah to define grassland polygons.

At the time of study, 23 prides were known to use the study area and were being intensively monitored by the SLP. Each pride is generally located using radio telemetry and observed directly at least once every 2 weeks. Unlike camera trap images, direct observation of lions allows for individual recognition from natural facial markings (Packer and Pusey 1993), and thus enables near-perfect knowledge of pride size and composition. In particular, the size of the female component of each study pride (excluding cubs) is known with a very high level of confidence. Our study does not consider a small number of transient nomadic females, which are known to remain in the study area for very short periods of time only (Packer et al. 2005).

Although a number of Serengeti prides restrict their activities exclusively to grassland habitat (*n* = 8), pride home ranges generally straddle both habitat types (*n* = 15). Failure to account for this is likely to lead to bias in habitat-specific female densities. To determine each pride’s contribution to female lion abundance in woodland and grassland habitats, we multiplied the known number of pride females by the proportion of the corresponding 75% home range that overlapped with either habitat type, which we denote as *p*. Since *p* is likely to vary seasonally for each pride owing to changing prey availability, we estimated seasonal 75% home ranges from pride-specific utilization distributions (UDs) drawn from the spatial coordinates of direct observations collected over the dry season of 2010 (Jun–Oct) and the wet season of 2011 (Nov–May), as per Mosser et al. (2009). Thus, for each pride, we obtained seasonal values of *p*, which we assumed reflected seasonal changes in the contribution to female lion abundance in woodland and grassland habitats. We estimated pride UDs using reference bandwidths (Silverman 1986) from functions implemented in the R package adehabitatHR (Calenge 2006). We defined 75% home ranges as the minimum area over which the probability of relocating a pride was equal to 0.75 (Calenge 2006, Mosser et al. 2009).

For both grassland and woodland habitats, we calculated seasonal reference density of female lions *D_ref_* as:

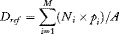
1in which *M* is the total number of lion prides with home ranges overlapping the habitat polygon, *N_i_* is the total number of female lions within pride *i* (with *i* ε M), *p_i_* is the proportion of pride *i*’s 75% home range overlapping the habitat polygon, and *A* is the habitat area effectively sampled by the camera trap grid. The latter was estimated by adding a buffer *w* equal to half the mean home range diameter of prides within the corresponding habitat to the camera trap grid hull (i.e., polygon obtained by joining the outer camera locations). We acknowledge that choice of buffer width for each habitat is the main source of uncertainty in our estimation of reference densities. To account for this, we derived reference density values for buffer widths corresponding to the 95% confidence limits of the corresponding habitat-specific mean home range radius. The resulting 95% confidence intervals surrounding our reference density values reflect the uncertainty associated with the estimation of *A*.

### REM Parameterization

We used the following REM equation to obtain density estimates from camera trap encounter rates (Rowcliffe et al. 2008: equation 4):

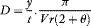
2in which *y* is the number of independent photographic events, *t* is total camera survey effort, *V* is average speed of animal movement, and *r* and *θ* are the radius and angle of the camera trap detection zone, respectively.

We considered only camera trap photographs taken in the dry season of 2010 (Aug–Oct) and the wet season of 2011 (Mar–May). We did not include images of male lions in our analyses owing to our reduced ability to accurately estimate reference male densities. We defined an independent contact with a camera as a female lion entering and exiting the field of view. Therefore, we considered consecutive photographic events of an individual lion remaining stationary in front of a camera as the same event. We calculated survey effort as the total number of camera hours, and obtained encounter rates by dividing the total number of independent photographic events of female lions by total survey effort. We defined night-time photographic events as those occurring between 1800 and 0600.

We carried out ex-situ field trials to determine the dimensions of the camera detection zone. To estimate camera radius *r*, we approached a test camera directly from the front and on all fours 10 times, and measured the distance from the camera to the location at first trigger for each approach. For camera angle *θ*, we carried out 10 paired approaches (1 from each side) perpendicular to the sensor beam at a distance of 5 m and recorded the location at first trigger. For each resulting location, we took a bearing using a compass placed on a flat surface directly below the camera. We recorded detection angle as the angle formed by the mean compass bearings taken on each side. We averaged values across trials to obtain values *r* and *θ*. We also carried out a sensitivity analysis to determine the effect of a 1% change in the value of *r* or *θ* on estimated density.

We estimated average distance moved per hour by female lions (hereafter, speed) from 4-day continuous follows of individual Serengeti prides carried out between September 1984 and December 1987 (see Packer et al. 1990, Scheel and Packer 1991). During these surveys, observers remained at least 200 m away from lions at all times. We derived distance moved from car odometer readings. For the purpose of this study, we assigned followed prides to woodland or grassland habitat based on the dominant habitat type within their 75% home range. For each season, we averaged hourly movement rate across prides of the same habitat to obtain average speed of movement during the 24-hour period (*V_all_*) and at night (*V_n_*).

Although lions are considered a social species and are often encountered as part of a pride, we chose not to define individual events as group contact events whereby REM density is multiplied by average group size (Rowcliffe et al. 2008, Zero et al. 2013). Our view is analogous to that put forward in the context of distance sampling of clustered animals. Thomas et al. (2010) acknowledge that treating grouped individuals as independent may sometimes be necessary if accurate group counts are not easily obtained, or if groups are not cohesive, as is the case for lions. In this case, variance surrounding the REM estimates will be inflated, but estimates remain unbiased (Thomas et al. 2010).

### Density Estimation

We defined 4 season-habitat subsets for which to estimate female lion densities: dry season-grassland (D-G), dry season-woodland (D-W), wet season-grassland (W-G), and wet season-woodland (W-W). We extracted habitat-specific camera points from corresponding habitat polygons (Fig. 1) and derived separate REM estimates from all and night-time only photographic events.

We computed overall variance of REM density estimates using the delta method (Seber 1982). The latter incorporated variance associated with the encounter rate (estimated by re-sampling camera locations with replacement 10,000 times, as per Rowcliffe et al. 2008), as well as standard errors associated with the independent estimation of parameters *V*, *r*, and *θ*. We used the resulting 95% confidence intervals to assess whether REM estimates differed significantly from reference values. We used percentage differences from reference densities to assess bias in REM estimates. We carried out all analyses in R version 3.0.3 (http://cran.r-project.org, accessed 2 Oct 2013).

## RESULTS

Prides with home ranges straddling both grassland and woodland habitat accounted for 65.2% and 60.9% of all monitored prides during the dry and the wet season, respectively (Table S1). Estimated reference female lion abundance was 102.7 for dry season-grassland, 47.2 for dry season-woodland, 112.3 for wet season-grassland, and 29.7 for wet season-woodland. Estimated survey area for grassland habitat was 824.9 km^2^ and 904.8 km^2^ during the dry and wet season, respectively. For woodland habitat, estimated surveyed area was 332.6 km^2^ and 358.6 km^2^ during the dry and wet season, respectively. Mean home range area was greatest in grassland habitat during the wet season (Table 1).

**Table:** Mean 75% pride home-range kernel area (HR; in km^2^) of lions in the Serengeti Lion Project study area during the dry season of 2010 and the wet season of 2011, and buffer width added to the habitat polygon for the different season-habitat combinations considered (*w*; in km)

Season-habitat^a^	No. of prides	Mean HR area	*w*	*w* 95% CI
D-G	13	49.1	4.0	2.6–5.2
D-W	10	46.6	3.9	2.8–5.3
W-G	13	78.0	5.0	3.5–6.2
W-W	10	51.6	4.1	2.2–5.0

a D-G, dry season-grassland; D-W, dry season-woodland; W-G, wet season-grassland; W-W, wet season-woodland.

We obtained 169 independent events of female lions from 7,608 camera trap days in the dry season, including 55 taken at night (see Table 2 for a summary of events recorded per season-habitat combination). For the wet season, 163 independent events were recorded over 12,137 camera trap days, including 73 at night. We used follow data from 3 woodland and 4 grassland prides to estimate habitat-specific average speed of lion movement in the dry and wet season, respectively (Table 2; Table S2). Average speed during both the 24-hour period and at night was highest in grassland habitat during the wet and dry seasons (Table 2). Estimates for camera detection radius and angle were 14.42 m (SE = 0.778 m) and 50.12° (SE = 1.557°), respectively. Our sensitivity analysis revealed that, all else remaining equal, a 1% change in detection radius resulted in a 1% change in density, whereas the same perturbation of the detection angle value only resulted in a 0.3% change in estimated density.

**Table:** Camera effort (in days) and number of independent photographic events of female Serengeti lions recorded in total and at night only for the different habitats considered during the dry season of 2010 and the wet season of 2011. *V_all_* and *V_n_* represent average speed of lion movement (in km/hr) over the 24-hour period and at night (± SE), respectively, and were derived from 96-hour continuous follows of selected prides

Season-habitat^a^	Effort	Total no. of events	No. night-time events	*V_all_*	*V_n_*
D-G	5,348	131	33	0.173 (±0.012)	0.287 (±0.017)
D-W	2,260	38	22	0.135 (±0.017)	0.275 (±0.020)
W-G	8,424	140	56	0.189 (±0.009)	0.307 (±0.013)
W-W	3,713	23	17	0.126 (±0.021)	0.288 (±0.028)

a D-G, dry season-grassland; D-W, dry season-woodland; W-G, wet season-grassland; W-W, wet season-woodland.

**Figure 2:**
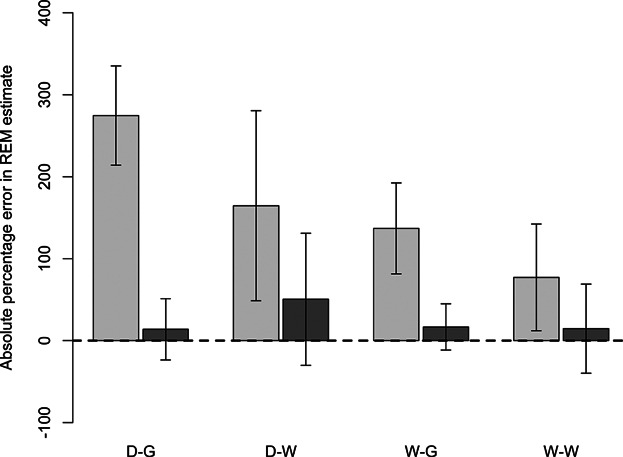
Absolute percentage errors associated with random encounter model (REM) estimates of female lion density in the Serengeti Lion Project study area during the dry season of 2010 and the wet season of 2011. Reference density is symbolized by the dashed horizontal line at 0% error. Bars represent errors in the estimates derived from all (light gray) or night-time only (dark gray) photographic events. Season-habitat combinations are defined as follows: dry season-grassland (D-G), dry season-woodland (D-W), wet season-grassland (W-G), and wet season-woodland (W-W). Error brackets represent the percentage errors of 95% confidence intervals associated with REM estimates.

All REM models considered over-estimated Serengeti female lion density but to varying degrees (Table 3; Fig. 2). Models that considered only night-time events resulted in estimates that were much less biased relative to those based on all photographic events. Estimates from all events were also significantly different from reference density values for all season-habitat combinations except in woodland habitat during the wet season. In contrast, confidence intervals associated with night-time REM estimates and reference densities overlapped for all season-habitat combinations. Although restricting data to night-time records had the strongest effect on accuracy, the effect of season was also notable, with wet season estimates based on all events being less biased than dry season estimates (Fig. 2). When considering all events, estimates from woodland habitat were closer to reference values in both seasons.

**Table:** Random encounter model (REM) and reference density (*D_ref_*) estimates (with 95% CI) for female Serengeti lions in grassland and woodland habitats during the dry season of 2010 and the wet season of 2011

	REM all events		REM night events		*D_ref_*	
Season-habitat^a^	Estimate	95% CI	Estimate	95% CI	Estimate	95% CI
D-G	0.467	0.391–0.542	0.142	0.094–0.190	0.124	0.111–0.141
D-W	0.375	0.211–0.539	0.213	0.096–0.330	0.142	0.111–0.170
W-G	0.294	0.222–0.366	0.145	0.109–0.181	0.124	0.112–0.142
W-W	0.147	0.093–0.201	0.095	0.050–0.139	0.083	0.072–0.120

a D-G, dry season-grassland; D-W, dry season-woodland; W-G, wet season-grassland; W-W, wet season-woodland.

## DISCUSSION

Estimates of animal density form the basis of many monitoring programs and often determine allocation of conservation efforts (Jones et al. 2013). Although camera traps offer a cost-effective way of gathering information on multiple species, methods for estimating density remain largely focused on marked species. In this context, the REM offers a simple framework that is potentially suited to a wider range of species. However, Foster and Harmsen (2012) suggest that the model’s assumption of random placement of cameras with respect to animal movement will often not be achievable for most species, and territorial large carnivores in particular. Countering this assessment, our study has shown that restricting REM estimation to periods and habitats in which animal movement is more likely to be random with respect to cameras can help reduce bias in estimates of density for female Serengeti lions. Nevertheless, we emphasize that despite this approach, our estimates remain biased to some degree in all season-habitat combinations, highlighting the need for truly random placement with respect to animal movement, as well as reliable estimates of average speed of animal movement and camera detection zone dimensions.

Lion movement in the Serengeti is primarily influenced by the distribution and density of prey (Hopcraft et al. 2005, Packer et al. 2005, Mosser and Packer 2009) but also at small scales by the distribution of landscape features, and trees in particular. The latter represent an important source of shade in a largely open savannah habitat. Thus, although the approximate locations of camera points across the study landscape were chosen using a systematic grid approach, preferential positioning of camera traps on trees at a finer scale represented a violation of the REM’s random placement assumption. However, using prior knowledge of lion behavior, we hypothesized that this violation would be less severe when tree cover is used less disproportionately by lions, specifically during the night, during the wet season, and in woodland habitat. By estimating densities using data filtered by these factors, we found that all 3 did indeed reduce bias, and most especially the exclusion of daytime records, which was alone sufficient to generate estimates that were not significantly different from reference in any habitat-season combination. In contrast, estimates obtained using both day and night-time data showed substantial and significant over-estimation in all cases. Given reliable estimates of average speed of animal movement and camera detection zone dimensions, these results highlight the capacity for the REM to provide unbiased density estimates for a large carnivore species, but only if the assumption of random distribution of cameras and animals relative to one another is met.

The REM performed well relative to other methods used to derive absolute lion densities in the past. Estimates were more precise (i.e., narrower confidence intervals) than those obtained for Serengeti lions using distance sampling (Durant et al. 2011), which also required a higher level of sampling effort. Similarly, the use of sight-resight methods to estimate lion density in Kenya’s Masai Mara was described by Ogutu et al. (2006) as “costly and time-consuming” owing to the necessity for accurate recognition of individual animals. In contrast, the REM may offer a promising and more cost-effective alternative to estimating animal density, provided model parameters are estimated accurately.

In line with this, a current drawback of the REM is its reliance on independent estimates of animal speed of movement and camera detection zone dimensions. Even in the case of the well-studied Serengeti lion population, estimates for these parameters still bear an unknown level of error. For example, camera sensitivity may be different for a 70-kg human than for a 200-kg lion. Although a method does exist that enables extraction of species-specific camera detection zone dimensions directly from the raw images (Rowcliffe et al. 2011), the camera model and settings used in this study precluded its application. Detection radius has been shown to decrease in the wet season, which could have affected REM estimates given that density is directly proportional to this parameter, as shown by our sensitivity analysis. In contrast, estimated density was found to be less sensitive to changes in camera detection angle.

For the same reason, obtaining accurate estimates of animal speed of movement is crucial to the model’s success. Despite this, studies using camera traps often possess limited information on the target species, including speed of movement. For example, Manzo et al. (2012) used day range estimates from Poland to estimate the density of European pine marten (*Martes martes*) in central Italy using the REM. Although we derived our estimates of average speed of lion movement from data collected nearly 30 years ago, there is no evidence that lion ranging patterns have changed in the interim. The intensive observations in the 1980s and the current camera trap survey were both conducted in the same general area, where there has been no substantial change in prey species (Packer et al. 2005, Sinclair et al. 2007) and human impacts have remained consistently low. Thus, given the scarcity of GPS collar data for Serengeti lions, we believe our estimates of speed to be adequate for the present study.

Our ability to assess the REM as a density estimation tool for Serengeti lions is dependent on reliable reference density values. Although we cannot exclude the possibility that a small number of itinerant females remain unaccounted for, our knowledge of female lion numbers in the study area is very close to complete because of intensive and on-going monitoring by the SLP. We acknowledge that defining the area sampled by the camera trap grid remains the primary source of uncertainty in our estimation of reference densities. However, we believe the range of buffer widths over which we estimated reference densities adequately reflects this uncertainty.

## MANAGEMENT IMPLICATIONS

Obtaining accurate estimates of animal density remains a constant challenge in the management and conservation of threatened large carnivores, and unmarked species in particular. We have shown that a relatively simple model, the REM, may be used to estimate the density of a territorial and unmarked large carnivore from camera trap data on the condition that clear violations of the model’s key assumption are identified and reduced as much as possible using prior knowledge of animal behavior. Indeed, the latter may be used to identify time periods (e.g., day or night) or camera locations (e.g., habitat type) that satisfy the requirement for randomness of cameras with respect to animals. Although we demonstrated this approach on existing camera trap data that were not collected with REM analysis in mind, we stress that, if available, knowledge of animal movement patterns should preferably guide REM survey design so as to avoid violations of the random placement assumption from the study onset. In the case of Serengeti lions, this suggests avoiding preferential positioning of camera traps on isolated trees. However, more generally for any species, we do not recommend application of the REM to data obtained from baited or lured cameras, from cameras placed preferentially on trails, watering points, mineral licks, outside of dens and known resting sites, or at any other landscape feature that may inflate or deflate capture rates. Nevertheless, our findings open up possibilities for the application of the REM to a broader range of unmarked species. In line with this, efforts are currently underway to streamline REM parameter estimation from the raw camera trap images (Rowcliffe et al. 2014), and it is hoped that these advances will greatly enhance standardization of the method as well as increase the accuracy of future estimates.

## Notes

Associate Editor: John Squires.

## Supporting Information

Supporting Information Table S1: Pride-specific information used to calculate season-habitat reference densities.

Supporting Information Table S2: Number of follow hours (in hours) and cumulative distance moved (in km) used to estimate average speed of lion movement.
