# Supplementary material for: Applying a random encounter model to estimate lion density from camera traps in Serengeti National Park, Tanzania
Source: J Wildl Manage. 2015 May 28;79(6):1014–21. doi: 10.1002/jwmg.902 (PMC4657488; doi:10.1002/jwmg.902)
Supplement: Supplementary file 1 [file jwmg0079-1014-sd1.docx]

Supplemental Material

14^th^ January 2015

Cusack et al. Applying a random encounter model to estimate lion density from camera trap data in Serengeti National Park, Tanzania. Journal of Wildlife Management: in review.

Table S1. Pride-specific information used to calculate season-habitat reference densities.

|  | 75% HR contour area | | Prop. HR in grassland | | Dominant habitat in HR | | Number of female lions^a^ | |
| --- | --- | --- | --- | --- | --- | --- | --- | --- |
| Pride code | Dry | Wet | Dry | Wet | Dry | Wet | Dry | Wet |
| BF | 106 | 118 | 0.34 | 0.74 | W | G | 8 | 7 |
| BH | 14 | 16 | 0.54 | 0.33 | G | W | 1 | 1 |
| CS | 24 | 40 | 0.49 | 0.56 | W | G | 4 | 5 |
| CV | 63 | 83 | 0.76 | 0.95 | G | G | 14 | 13 |
| EP | 56 | 95 | 1.00 | 1.00 | G | G | 2 | 2 |
| JK | 29 | 44 | 1.00 | 1.00 | G | G | 3 | 3 |
| KB | 29 | 66 | 0.29 | 0.49 | W | W | 6 | 7 |
| LL | 49 | 79 | 0.21 | 0.28 | W | W | 8 | 7 |
| MH | 82 | 73 | 0.76 | 0.93 | G | G | 3 | 1 |
| MK | 51 | 78 | 0.89 | 0.90 | G | G | 11 | 9 |
| MM | 42 | 130 | 0.76 | 0.88 | G | G | 3 | 3 |
| MY | 26 | 35 | 0.77 | 0.81 | G | G | 6 | 7 |
| N3 | 50 | 44 | 1.00 | 1.00 | G | G | 2 | 2 |
| PN | 55 | 88 | 1.00 | 1.00 | G | G | 8 | 10 |
| S# | 24 | 45 | 1.00 | 1.00 | G | G | 18 | 17 |
| SB | 73 | 73 | 1.00 | 1.00 | G | G | 6 | 7 |
| SP | 34 | 92 | 0.96 | 0.98 | G | G | 3 | 3 |
| SS | 34 | 116 | 1.00 | 1.00 | G | G | 13 | 9 |
| SU | 55 | 106 | 1.00 | 1.00 | G | G | 3 | 3 |
| TR | 25 | 61 | 0.14 | 0.22 | W | W | 9 | 7 |
| TT | 43 | 45 | 0.20 | 0.36 | W | W | 3 | 3 |
| VU | 97 | 65 | 0.85 | 1.00 | G | G | 5 | 5 |
| YT | 50 | 80 | 0.02 | 0.48 | W | W | 10 | 10 |

^a^Excluding cubs.
