# Supplementary material for: Applying a random encounter model to estimate lion density from camera traps in Serengeti National Park, Tanzania
Source: J Wildl Manage. 2015 May 28;79(6):1014–21. doi: 10.1002/jwmg.902 (PMC4657488; doi:10.1002/jwmg.902)
Supplement: Supplementary file 2 [file jwmg0079-1014-sd2.docx]

Supplemental Material

14^th^ January 2015

Cusack et al. Applying a random encounter model to estimate lion density from camera trap data in Serengeti National Park, Tanzania. Journal of Wildlife Management: in review.

Table S2. Number of follow hours (in hours) and cumulative distance moved (in km) used to estimate average speed of lion movement. Data was collected during 4-day continuous follows of individual Serengeti prides carried out between September 1984 and December 1987 (see Packer et al. 1990, Scheel & Packer 1991). Distance moved was derived from car odometer readings. For the purpose of this study, followed prides were assigned to woodland or grassland habitat based on the dominant habitat type within their 75% home range. Values associated with night-time periods are given in parentheses.

| Habitat | Pride code | Number of follow hours | |  | Cumulative distance moved | |
| --- | --- | --- | --- | --- | --- | --- |
|  |  | Dry season | Wet season |  | Dry season | Wet season |
| Woodland | KB | 96 (48) | 36 (14) |  | 14.6 (12.2) | 4.5 (3.6) |
|  | CS | 96 (36) | 96 (36) |  | 11.3 (9.9) | 14.1 (10.4) |
|  | LL | 72 (30) | 72 (24) |  | 9.7 (8.9) | 8.3 (7.6) |
| Grassland | SP | 96 (48) | 96 (48) |  | 16.6 (13.8) | 18.1 (14.7) |
|  | PN | 96 (48) | - |  | 15.5 (13.0) | - |
|  | SB | 96 (48) | 96 (48) |  | 17.8 (14.6) | 19.0 (15.4) |
|  | SU | - | 96 (48) |  | - | 17.3 (14.1) |
